# Reliability of quantitative and qualitative chorioretinal uveitis lesion analysis on blue, green, and near-infrared fundus autofluorescence and color fundus photography

**DOI:** 10.1038/s41598-026-54450-y

**Published:** 2026-06-01

**Authors:** Marie D. Just, Jana Koch, Gabriela Guzman, Lennart J. Overbeck, Moritz Berger, Jan H. Terheyden, Selina Foti, Matthias Schmid, Frank G. Holz, Marlene Saßmannshausen, Matthias M. Mauschitz, Robert P. Finger, Maximilian W. M. Wintergerst

**Affiliations:** 1 Department of Ophthalmology, University Hospital Bonn, University of Bonn, Ernst-Abbe-Straße 2, 53127 Bonn, Germany; 2 Department of Medical Biometry, Informatics and Epidemiology, Medical Faculty, University of Bonn, Bonn, Germany; 3 Core Facility Biostatistics, Medical Faculty Mannheim, Central Institute of Mental Health, Heidelberg University, Mannheim, Germany; 4 Department of Ophthalmology, University Medical Center Mannheim, Heidelberg University, Mannheim, Germany; 5 Augenzentrum Grischun, Chur, Switzerland

**Keywords:** Posterior uveitis, Fundus autofluorescence, Color fundus photography, Inter-rater reliability, Quantitative imaging biomarkers, Endpoint, Biomarkers, Diseases, Medical research

## Abstract

As there is a lack of reliable structural outcome parameters for posterior uveitis trials, we compared inter-rater reliability (IRR) of chorioretinal lesion area measurement (quantitative analyses) and characterization (qualitative analyses) on color fundus photography (CFP) and multimodal fundus autofluorescence (FAF). In this prospective cohort study, posterior uveitis eyes were imaged with CFP (Eidon, iCare, Vantaa), short- (swBAF, 450 nm; Eidon) and long-wavelength blue-light-autofluorescence (lwBAF, 488 nm), green-light-autofluorescence (GAF, 518 nm), and infrared-autofluorescence (IRAF, 787 nm) (all Spectralis, Heidelberg Engineering, Heidelberg). Lesion area, measured with ImageJ (National Institutes of Health), and image characteristics of lesions were graded on all modalities by two masked raters. A total of 318 lesions from 27 eyes (17 patients) were included. Absolute inter-rater differences in area measurement were 0.57, 0.78, 0.30, 0.36, and 0.55 in standardized 10^3^ pixels for CFP, swBAF, lwBAF, GAF, and IRAF, respectively. IRR was high for all modalities for quantitative (intraclass correlation, 95% confidence interval (CI) [0.997–0.998]) and at least substantial for qualitative measures (unweighted Cohen’s kappa 0.84, 0.91, 0.89, 0.91, and 0.89 for CFP, swBAF, lwBAF, GAF, and IRAF, respectively, all *p* < 0.0001, CIs [0.79–0.95]). Hence, FAF could be a reliable complementary imaging modality for posterior uveitis clinical trials, especially for lesion quantification.

## Introduction

Posterior uveitis encompasses a spectrum of diseases involving inflammation of the posterior eye segment, affecting the choroid, retina, and retinal vasculature. Posterior uveitis may have poor prognosis if inadequately treated. It often requires long-term immunomodulatory therapy and up to a third of patients develop severe visual impairment^1–5^. As younger patients are frequently affected, consequences are often severe and may lead to unemployment, reduced quality of life and depression^1,4^. As posterior uveitis is a rare disease and comprises very different entities, there is only little high-level evidence available regarding specific management^3,6,7^. Pre-requisites for high-level evidence are randomized controlled clinical trials (RCTs). For any trials to be implemented, reliable, standardized and validated endpoints relevant to the specific research question and assessed disease are needed^8^. No such endpoints are available for posterior uveitis, which negatively affects the implementation of RCTs and generation of high-level evidence^8^. Thus, novel, robust endpoints need to be developed. While standardization and likely reliability of clinical assessment have improved with the ‘Standardization of Uveitis Nomenclature’ (SUN), evidence for reliability of different image modalities for assessment of posterior uveitis are lacking^9,10^. Hence, there is a need to assess reliability of quantifiable imaging biomarkers for posterior uveitis, which could be used as clinical trial endpoints^4,10^.

Reliable monitoring of lesion size and, therefore, disease progression is crucial for the therapeutic management as well as for RCTs. The current approach relies on assessments of color fundus photography (CFP), however evidence suggests that its sensitivity is limited, as not all retinal lesions are detectable on CFP^11–15^. Fluorescein (FLA) and indocyanine green angiography (ICG) are helpful imaging modalities, however, their invasive nature limits regular and frequent examinations.

Fundus autofluorescence (FAF) has emerged as a promising approach for evaluation of inflammatory retinal lesions in posterior uveitis, as inflammatory activity is linked to appearance on FAF: newly developed inflammatory lesions of several posterior uveitis syndromes exhibit hyperautofluorescence on FAF, while areas of quiescent disease are hypoautofluorescent^16^. Furthermore, FAF aids to reveal inflammatory lesions not visible on ophthalmoscopy or by CFP^11–15^.

FAF has been suggested for monitoring of posterior uveitis due to its prognostic and therapeutic value^11–15,17–28^. To date, there is no consensus on the FAF modality to be used in posterior uveitis. The excitation wavelength varies from 450 to 787 nm depending on the FAF device and study, e.g. 488 nm excitation wavelength and a barrier filter set at 500 nm (Heidelberg HRA)^15,26,29,30^, 560 nm (bandwidth, 535–585 nm) excitation wavelength and a barrier filter set at 655 nm (bandwidth, 605–705 nm) (Topcon Medical Systems, Paramus, NJ)^11,31^, 585 nm (bandwidth 550–600 nm) excitation wavelength and a barrier filter set at 690 nm (Topcon)^32,33^. In a few studies, more than one FAF modality were used to assess uveitic chorioretinal lesions, namely conventional blue-light-autofluorescence (488 nm, Heidelberg HRA) and infrared-autofluorescence (787 nm)^28,34^ or green-light-autofluorescence (532 nm)^35^. Inflammatory lesions in posterior uveitis were shown to vary considerably between different FAF modalities depending on excitation wavelength^16^ Further, spectrally resolved autofluorescence imaging can reveal varying green and red emission fluorescent components in different posterior uveitis entities^36^.

However, reliability of chorioretinal lesion size quantification and analysis of lesion characteristic on CFP and FAF are unclear, albeit essential for use as a feasible clinical endpoint^10^. Thus, we assessed and compared reliability of standardized chorioretinal lesion size quantification and their characterization on CFP and different FAF modalities.

## Methods

### Subject recruitment

Approval (ID 011/18) was obtained from the ethics committee of the University of Bonn. The study was conducted according to the tenets of the Declaration of Helsinki. Informed consent was obtained from all patients before enrollment. Patients with posterior uveitis and panuveitis according to the diagnostic criteria defined by the SUN working group^9^ were recruited from the outpatient clinic at the Department of Ophthalmology at the University of Bonn, Bonn, Germany, and enrolled in the study. Adult patients (≥ 18 years) able to perform CFP, swBAF, lwBAF, GAF, and IRAF imaging were eligible for the study. We excluded all patients with reduced image quality on CFP or FAF, a history of diabetes mellitus or any other retinal diseases beside uveitis, corneal, lens or vitreous opacities impairing image quality, or ocular surgery except cataract surgery.

### Image acquisition

All patients underwent pupil dilatation (using 0.5% tropicamide, 2.5% phenylephrine) and were examined by an uveitis trained ophthalmologist. Activity of Uveitis was graded according to Standardized Uveitis Nomenclature (SUN)^9^. Vitreous inflammation was graded as “present” if Haze was at least + 1 according to SUN or vitreous cells were present (subjective clinical grading at least + 1) and otherwise as “absent”. Eyes were imaged by confocal laser scanning CFP (Eidon, iCare Finland Oy, Vantaa, Finland; formerly CenterVue) and multimodal FAF, including short-wavelength blue-light-autofluorescence (swBAF, 450 nm) (Eidon) and long-wavelength blue-light-autofluorescence (lwBAF, 488 nm), green-light-autofluorescence (GAF, 518 nm), and infrared-autofluorescence (IRAF, 787 nm) (Spectralis HRA, Heidelberg Engineering GmbH, Heidelberg, Germany). Spectralis HRA FAF images were acquired in high-resolution (HR) mode, with the highest possible Automatic Real-time Tracking (ART) value (max. 100). Based on the retinal lesion’s location images were mainly centered at the fovea or optic nerve head, or temporal from the macula. Image resolutions were 3680 × 3288 pixels (Eidon) or 1536 × 1536 pixels (Spectralis HRA). If multiple images were available, images with the best image quality were selected for analyses.

### Image analysis

Two masked raters (doctoral students supervised by an ophthalmologist specializing in the field of uveitis) analysed the images quantitatively and qualitatively on a 27-inch full HD monitor with a resolution of 1920 × 1080 pixels in a dimmed light environment. To structure the image data, all images were exported and processed with the Adobe Photoshop (CS2 Version 9.0; Adobe Systems Software Ireland Limited, Dublin, Ireland) and facilitated the comparison within an eye by switching between overlaid images of all image modalities. The first step of the analysis was to determine and quantify which lesions were visible in the different modalities. Next, the chorioretinal lesions were numbered. Lesions that were not clearly identifiable as one continuous lesion were discussed between the raters to ensure that the lesion number matched. Lesions not fully visible on all image modalities (e.g. due to extending over the border of the image, covered by vitreous opacities or insufficiently illuminated) were excluded from the analyses.

For qualitative analysis images were viewed on a set zoom scale of 25% using Adobe Photoshop (CS2 Version 9.0). Images were viewed without knowledge of the previously assigned lesion numbers. CFP images were analyzed first to avoid reader bias by previously seen lesions on FAF that are invisible on CFP. Afterwards, the lesions were categorized by their appearance. In CFP images, the following categories were utilized: dark, not visible, bright, complex (complex lesions needed to have at least one dark and one bright part), and slightly visible (greyish / brownish). In FAF images, the categories were hypoautofluorescent, isoautofluorescent, hyperautofluorescent and complex (defined as both hypo- and hyperautofluorescent).

**Fig. 1:**
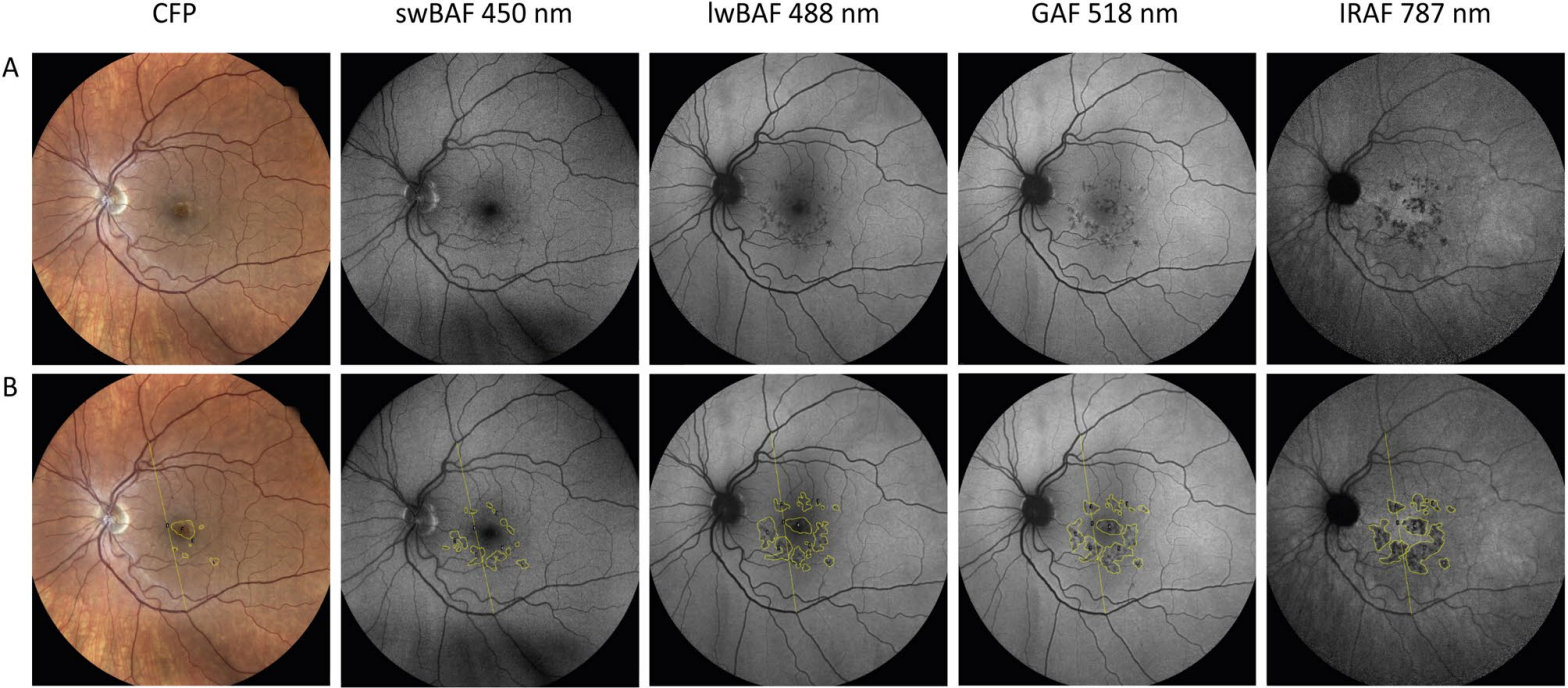
Measurement of chorioretinal lesion size on color fundus photography (CFP) and different fundus autofluorescence (FAF) modalities. Multimodal imaging of left eye of patient with acute posterior multifocal placoid pigment epitheliopathy. (**A**) native images, (**B**) images with overlay of the eye-specific scale (straight line connecting branching points in the vasculature of the upper and lower temporal arcade), numbered lesion and lesion size measurements. The eye was imaged with CFP (Eidon, iCare), short-wavelength blue-light-autofluorescence (swBAF,450 nm; Eidon), long-wavelength blue-light-autofluorescence (lwBAF, 488 nm), green-light-autofluorescence (GAF, 518 nm), and infrared-autofluorescence (IRAF, 787 nm; all Spectralis, Heidelberg Engineering).

Quantitative assessment of lesion size was performed using Fiji^37^(an expanded version of ImageJ (National Institutes of Health, Bethesda, Maryland, USA; available at “https://imagej.nih.gov/ij/”)^38^. The zoom scale was set to 100% (Eidon images) and 200% (Spectralis HRA images) to account for the different image resolution. For analyses of very small lesions, zoom was increased to 150% (Eidon images) and 300% (Spectralis HRA images), respectively. A scale was defined for all images from one eye for standardization of quantitative measurements, allowing comparison of measurements from different image resolutions. The scale was defined as the connection of two easily identifiable branching points in the vasculature of the upper and lower temporal arcade (see Fig. 1). The lesion borders were drawn using the “freehand selection” tool in Fiji (see Fig. 1). The marked areas were measured in pixels and were saved as Region of interest (ROI). Lesions that were not visible on CFP were given an area of 0 pixels, for better comparability, subsequently all imaging modalities of these specific lesions were excluded from the statistical analysis. This resulted in a slightly lower number of lesions in the quantitative compared to the qualitative analysis.

### Statistical analysis

Statistical analyses were performed using R version 3.4.1^39^. Absolute differences in lesion area measurements between the two raters were determined for each imaging modality. Using the standardized scale, absolute measurements were normalized, allowing comparison between image modalities with different image resolutions. Differences in lesion area measurements were statistically analysed using the Kruskal-Wallis rank sum test with a significance level (α) of 0.05^40^. Inter-rater reliability of quantitative assessment of lesion size in CFP and all FAF modalities between two raters was calculated using the intraclass correlation coefficient (ICC) (model = “two-way”, type = “consistency”) with a 95% cnfidence interval. ICC is used to measure the reliability of a rating by calculating the proportion of the total variance in the ratings due to differences between the raters and the proportion due to differences between the rated subjects. ICC values range from 0 to 1, with higher values indicating greater reliability^41^. In this study, a two-way model ICC was used. Based on the confidence interval, ICC values below 0.5 were considered to indicate poor reliability, values between 0.5 and 0.75 were considered to indicate moderate reliability, values between 0.75 and 0.9 were considered to indicate good reliability, and values above 0.9 were considered to indicate excellent reliability^42^. Inter-rater reliability of qualitative lesion assessment was calculated using the unweighted Cohen’s kappa (κ) (α = 0.05) with a 95% confience interval. Inter-rater reliability for categorical variables can be measured using Cohen’s kappa. It considers the agreement that might occur by chance and returns a value between − 1 and 1, with a value of 1 indicating perfect agreement, a value of 0 indicating no better agreement than chance, and negative values indicating less agreement than chance. k values were interpreted using the following threshold: < 0.20 is considered poor agreement, 0.21 to 0.40 is considered fair agreement, 0.41 to 0.60 is considered moderate agreement, 0.61 to 0.80 is considered substantial agreement, and > 0.80 is considered almost-perfect agreement^43–45^.

## Results

### Demographics

Twenty-seven eyes of 17 patients (10 females, 7 males, mean age = 49 ± 19 years) with posterior uveitis (24 eyes) and panuveitis (3 eyes) were included in the study. Of these, 9 eyes were in remission, 8 eyes were inactive, 2 showed worsening and 8 improved activity of uveitis. Vitreous inflammation was present in 8 eyes. One eye was excluded from the quantitative analysis due to an image being partially non-evaluable. No patients were excluded due to overall insufficient image quality. Demographics and uveitis entities are provided in Table 1. A total of 318 chorioretinal lesions were quantified for lesion size and were qualitatively analyzed for lesion characteristics (on average 12.56 ± 12.26 lesions / eye). In total, 74 lesions that were not fully visible on all imaging modalities as described above were excluded.

**Table 1:** Characteristics of the sample.

	Mean (± SD) or *n* (%)
Age (years)	49 (± 19)
Sex	
Female	10 (58.8)
Male	7 (41.2)
Uveitis entities (eyes)	
Idiopathic	12 (44.4)
Ampiginous choroiditis	4 (14.8)
APMPPE	4 (14.8)
MEWDS	2 (7.4)
PIC	2 (7.4)
Serpiginous choroiditis	2 (7.4)
Multifocal choroiditis	1 (3.7)
Activity of uveitis*	
Inactive	8 (29.6)
Worsening activity	2 (7.4)
Improved activity	8 (29.6)
Remission	9 (33.3)
Vitreous inflammation**	
Absent	19 (70.4)
Present	8 (29.6)

SD, standard deviation; n, number; APMPPE, acute posterior multifocal placoid pigment epitheliopathy; MEWDS, multiple evanescent white dot syndrome; PIC, punctate inner choroiditis; *Activity of Uveitis was graded according to Standardized Uveitis Nomenclature (SUN)^9^; **Vitreous inflammation was graded as “present” if Haze was at least + 1 according to SUN or vitreous cells were present (subjective clinical grading at least + 1) and otherwise as “absent”.

### Quantitative and qualitative lesion analysis on CFP and FAF modalities

Inter-rater reliability (ICC [95% CI]; all *p* < 0.0001) of lesion area measurements showed an excellent reliability for CFP (0.998 [0.997–0.998]), swBAF (0.999 [0.999–0.999]), lwBAF (0.999 [0.999–1.00]), GAF (0.999 [0.999–1.00]), and IRAF (0.998 [0.997–0.998]), hence turned out not to be suitable for quantifying the observed modality-dependent differences. Overall, area measurement of chorioretinal lesions in uveitis were more reliable on lwBAF and GAF as compared to CFP, IRAF and swBAF. Inter-rater reliability of lesion characterization demonstrated a substantial to almost-perfect agreement across all modalities (swBAF, lwBAF, GAF, IRAF and CFP) (Table 2). Qualitative lesion characterizations were reliable on all modalities, the distribution of qualitative lesion analyses across categories of imaging modalities is shown in Table 3. Most of the lesions were not visible or only slightly visible on CFP. Overall on FAF most of the lesions were characterized as hypoautofluorescent, but distribution varies between swBAF, lwBAF, GAF, and IRAF.

**Table 2:** Reliability of quantitative and qualitative lesion analysis on color fundus photography and fundus autofluorescence modalities.

Imaging modality	Quantitative assessment	Qualitative characterization
	Differences in area measurement in standardized 10^3^ pixels Mean ± SD; Median; [IQR]	Unweighted kappa (κ) [95% CI]
Color fundus photography (CFP)	Mean 4.37 ± 15.22 Median 0.57 [− 0.685–1.825]	0.84 [0.79–0.89]
Short-wavelength blue-light-autofluorescence (swBAF, 450 nm)	Mean 2.98 ± 9.68 Median 0.78 [− 0.45–2.01]	0.91 [0.87–0.94]
Long-wavelength blue-light-autofluorescence (lwBAF, 488 nm)	Mean 1.18 ± 3.38 Median 0.30 [− 3.55–4.15]	0.89 [0.85–0.93]
Green-light-autofluorescence (GAF, 518 nm)	Mean 1.31 ± 3.55 Median 0.36 [− 0.105–0.825]	0.91 [0.86–0.95]
Infrared-autofluorescence (IRAF, 787 nm)	Mean 2.28 ± 7.76 Median 0.55 [− 0.15–1.25]	0.89 [0.84–0.93]

Differences in lesion area measurements were determined by calculating the mean and median of lesion measurements in each imaging modality using the Kruskal-Wallis rank sum test (*p* < 0.0001, IQR [25% Quartile – 75% Quartile]). Inter-rater agreement of qualitative lesion characterization was calculated using unweighted Cohen´s kappa (κ) (*p* < 0.0001, [95% CI]). SD,   standard deviation; IQR , interquartile range; CI, confidence interval.

**Table 3:** Qualitative lesion analysis on color fundus photography and fundus autofluorescence modalities.

Color fundus photography (CFP)						
	Not visible (%)		Slightly visible (%)	Dark (%)	Bright (%)	Complex (%)
Grader 1	33.53		28.49	0.59	26.11	11.28
Grader 2	35.61		28.78	0.59	25.52	9.50

Fundus autofluorescence (FAF)						
			Hypoautofluorescent (%)	Isoautofluorescent (%)	Hyperautofluorescent (%)	Complex (%)
Short-wavelength blue-light-autofluorescence (swBAF, 450 nm)		Grader 1	32.73	25.53	16.52	25.23
		Grader 2	32.43	25.83	16.82	24.92
Long-wavelength blue-light-autofluorescence (lwBAF, 488 nm)		Grader 1	45.92	19.94	4.83	29.31
		Grader 2	43.81	21.45	3.32	31.42
Green-light-autofluorescence (GAF, 518 nm)		Grader 1	48.04	19.34	2.11	30.51
		Grader 2	47.13	20.24	2.11	30.51
Infrared-autofluorescence (IRAF, 787 nm)		Grader 1	61.47	9.17	1.22	28.13
		Grader 2	61.77	10.09	0.92	27.22

Distribution of qualitative lesion analyses across categories of imaging modalities.

## Discussion

Our study demonstrates that inter-rater reliability for quantitative analysis of chorioretinal lesion size in posterior uveitis was superior on lwBAF and GAF compared to CFP, swBAF, and IRAF. Furthermore, reliability for analysis of qualitative lesion characteristics was almost perfect on all previous mentioned modalities. These results indicate a higher reliability for quantitative structural posterior uveitis endpoints on most FAF modalities compared to CFP. For qualitative structural posterior uveitis endpoints, both FAF modalities and CFP are highly suitable in terms of reliability. Although current recommendations do not highlight FAF as a universal requirement, it may still represent a valuable diagnostic modality. In 2025, the Multimodal Imaging (MUV) Task Force^46^ proposed a minimum imaging set for the diagnosis, activity monitoring, and complication assessment of noninfectious posterior uveitis. Within this framework, FAF was considered essential for both diagnosis and activity evaluation in multiple evanescent white dot syndrome (MEWDS) and serpiginous choroiditis (SC). In contrast, FAF was not regarded as essential for acute posterior multifocal placoid pigment epitheliopathy (APMPPE), birdshot chorioretinopathy (BSCR), or multifocal choroiditis with panuveitis/punctate inner choroidopathy (MFCPU/PIC). Notably, the Task Force did not distinguish between different FAF excitation wavelengths.Our study emphasizes the large benefits of including structural FAF endpoints in future RCTs for posterior uveitis. Furthermore, monitoring of disease in routine clinical practice may benefit from FAF imaging.

The use of FAF imaging has already been evaluated for different entities of uveitis^16^, but, to the best of our knowledge, this is the first study comparing CFP and different FAF modalities in terms of inter-rater reliability in patients with posterior uveitis. FAF enables the detection of chorioretinal lesions not visible on standard CFP as it provides deeper insights into the extent of RPE damage and consequently early metabolic alterations, and molecular changes^11,13,20,47.^ Moreover, it can aid in distinguishing between active and inactive areas within inflammatory lesions^11,48.^ Although CFP has long been an important tool for documenting the course of disease in patients with inflammation of the posterior segment of the eye^49^, relying only on CFP may result in misinterpretation of lesion size or activity and should therefore be complemented by additional imaging modalities.

The differences observed in the quantitative analysis of the present study may also be related to the hardware specifications of the different imaging devices (Eidon [3680 × 3288 pixels] for CFP and swBAF versus Spectralis [1536 × 1536 pixels] for lwBAF, GAF, IRAF). A possible explanation for the less reliable lesion area measurement on swBAF compared to lwBAF and GAF might also be the different penetration depth of the shorter wavelength in swBAF. Further, a wavelength of 450 nm appears to excite a different range of fluorophores, resulting in a respectively low and rather diffuse emission signal that could compromise the device’s capacity to detect and assess the lesion area^50.^ For IRAF, the reduced reliability is possibly due to a lower contrast of the lesions compared to the rather heterogeneous background autofluorescence in IRAF (in contrast to lwBAF and GAF, choroidal vessels are seen on IRAF, which can make it difficult to precisely delineate chorioretinal lesions). ICC does not appear to be a suitable parameter to analyse inter-rater reliability for lesion size measurements in uveitis, as reliability of all modalities was excellent in terms of ICC and the differences observed in the absolute quantitative measurements were not reflected. Consistent with our findings, for well-defined diseases such as age-related macular degeneration, ICC for geographic atrophy area was also high for CFP (0.96 [0.94–0.97]) and for lwBAF (0.99 [0.98–0.99])^51.^ The minor differences observed in the qualitative analysis may be related to the different number of possible classifications: while 5 options were available for CFP (dark, not visible, bright, complex, and slightly visible), only 4 options were available for FAF (hypoautofluorescent, isoautofluorescent, hyperautofluorescent and complex). Given the additionally provided and yet not fully understood phenotypic information on FAF in posterior uveitis, these additional imaging biomarkers might also aid in predicting prognosis. This, however, cannot be analyzed in a cross-sectional setting.

The strengths of our study include the systematic standardized comparison of a variety of different FAF modalities with CFP, the prospective nature, use of established, commercially available non-invasive imaging techniques, stringent inclusion and exclusion criteria, and a large number of included chorioretinal lesions.

The limitations of our study are a relatively small sample size in terms of eyes and the relatively heterogenous sample in terms of posterior uveitis entities. However, given the rarity of the disease, a heterogeneous disease spectrum of posterior uveitis and panuveitis had been included to allow a comprehensive analysis. Second, the lack of longitudinal imaging may limit direct conclusions for patient monitoring. However, as our aim was to assess inter-rater reliability in the different imaging modalities, a cross-sectional design was sufficient. Future longitudinal studies will be valuable to establish applicability for patient monitoring. Third, we did not stratify between active and inactive disease. To avoid bias, we excluded lesions that were cut off by image borders, obscured by vitreous opacities, or in poorly illuminated areas. These exclusions were technical rather than related to disease severity, ensuring that reduced image quality from inflammation did not influence the results significantly.

The different number of optional grading classifications also represents a limitation of our study. However, this discrepancy is due to the fact that all observed image characteristics were taken into account as precisely as possible. Combining several categories would have biased the accuracy of the observations. Further, we did not analyze possible differences in green and red emission fluorescent components, although this ratio can be significantly different between distinct entities^36.^ Subgroup analyses and the differentiation of uveitis entities are warranted, but challenging to conduct due to the rarity of the diseases. Another possible subgroup analysis would be the consideration of lesion localization, as GAF allows foveal imaging without macular pigment interference compared to BAF^35.^ Yet, preliminary analyses of our data revealed no significant differences. Given possible differences in image quality between devices, comparison of FAF devices from more than two devices would have been interesting, but, would have largely extended our already comprehensive imaging protocol.

Our study emphasizes the potential additional value of FAF imaging in patients with posterior uveitis to achieve higher reliability in quantitative analysis of chorioretinal lesions. Both in the clinical as well as in the study setting, an accurate assessment of lesion progression is essential. Within the FAF modalities investigated, lwBAF, and GAF demonstrated superior reliability compared to swBAF, IRAF, and CFP in terms of lesion area measurement. Reliability of lesion characterization was almost perfect on all FAF modalities and CFP. This should be considered when selecting endpoints for posterior uveitis RCTs, as FAF might represent a viable complementary image modality providing reliable structural outcome parameters for posterior uveitis. Larger studies on specific posterior entities and longitudinal analyses to compare different FAF modalities for their value in monitoring disease and possible prediction of prognosis are warranted.

## Data Availability

Data is available upon reasonable request. For inquiries, please contact the corresponding author.
